# Serological surveillance of GI norovirus reveals persistence of blockade antibody in a Jidong community-based prospective cohort, 2014–2018

**DOI:** 10.3389/fcimb.2023.1258550

**Published:** 2023-12-18

**Authors:** Jing-Rong Yu, Dong-Jie Xie, Jia-Heng Li, Mark Momoh Koroma, Lu Wang, Yu Wang, Duo-Na Jing, Jia-Yi Xu, Jun-Xuan Yu, Hui-Sha Du, Fei-Yuan Zhou, Zhi-Yan Liang, Xu-Fu Zhang, Ying-Chun Dai

**Affiliations:** ^1^ Department of Epidemiology, Guangdong Provincial Key Laboratory of Tropical Disease Research, School of Public Health, Southern Medical University, Guangzhou, China; ^2^ Department of Public Health, Shenzhen Qianhai Shekou Free Trade Zone Hospital, Shenzhen, China; ^3^ The Fifth Affiliated Hospital, Southern Medical University, Guangzhou, China; ^4^ School of Traditional Chinese Medicine, Southern Medical University, Guangzhou, China

**Keywords:** GI norovirus, dynamics, blockade antibody, serological surveillance, HBGA

## Abstract

**Introduction:**

Herd immunity against norovirus (NoV) is poorly understood in terms of its serological properties and vaccine designs. The precise neutralizing serological features of genotype I (GI) NoV have not been studied.

**Methods:**

To expand insights on vaccine design and herd immunity of NoVs, seroprevalence and seroincidence of NoV genotypes GI.2, GI.3, and GI.9 were determined using blockade antibodies based on a 5-year longitudinal serosurveillance among 449 residents in Jidong community.

**Results:**

Correlation between human histo-blood group antigens (HBGAs) and GI NoV, and dynamic and persistency of antibodies were also analyzed. Seroprevalence of GI.2, GI.3, and GI.9 NoV were 15.1%–18.0%, 35.0%–38.8%, and 17.6%–22.0%; seroincidences were 10.0, 21.0, and 11.0 per 100.0 person-year from 2014 to 2018, respectively. Blockade antibodies positive to GI.2 and GI.3 NoV were significantly associated with HBGA phenotypes, including blood types A, B (excluding GI.3), and O^+^; Lewis phenotypes Le^b+^/Le^y+^ and Le^a+b+^/Le^x+y+^; and secretors. The overall decay rate of anti-GI.2 antibody was -5.9%/year (95% CI: -7.1% to -4.8%/year), which was significantly faster than that of GI.3 [-3.6%/year (95% CI: -4.6% to -2.6%/year)] and GI.9 strains [-4.0%/year (95% CI: -4.7% to -3.3%/year)]. The duration of anti-GI.2, GI.3, and GI.9 NoV antibodies estimated by generalized linear model (GLM) was approximately 2.3, 4.2, and 4.8 years, respectively.

**Discussion:**

In conclusion, enhanced community surveillance of GI NoV is needed, and even one-shot vaccine may provide coast-efficient health benefits against GI NoV infection.

## 1 Introduction

Noroviruses (NoVs) are the leading cause of outbreaks and sporadic cases of acute gastroenteritis (AGE) globally, affecting people of all ages (van Beek et al., 2018). They are highly contagious and are usually spread from person to person from contaminated food, water, and environment through the fecal–oral route and aerosol transmission (Atmar, 2010). NoV causes a significant disease burden worldwide, mainly in developing countries, with an estimated 690 million cases of diarrhea and nearly 220,000 fatalities annually, exhibiting a substantial global public health concern of NoVs (Kirk et al., 2015).

The genome of NoV is divided into three open reading frames (ORFs), among which ORF2 encodes the major capsid protein (VP1) (Thorne and Goodfellow, 2014; Deval et al., 2017; Ludwig-Begall et al., 2021). VP1 is divided into the shell (S) domain and the protruding (P) domain, which is the main antigenic determinant and receptor-binding domain (Thorne and Goodfellow, 2014). Due to their genetic and antigenic diversity, NoVs are classified into 10 genogroups (GI–GX) and 48 genotypes, with GI and GII being the most predominant cause of NoV infections (Chhabra et al., 2019).

Human histo-blood group antigens (HBGAs) are thought to be the primary receptors or coreceptors for the NoVs. HBGAs are a group of glycoantigens with a high variety of polymorphisms, which consist of three main categories: ABO blood types (A, B, AB, O^+^, and O^-^), Lewis phenotypes (Le^a+^/Le^x+^, Le^b+^/Le^y+^, and Le^a+b+^/Le^x+y+^), and Secretor status (secretor and nonsecretor) (Kambhampati et al., 2016; Chassaing et al., 2020). NoVs use glycans of the ABH and Lewis family for attachment (Tan and Jiang, 2014). It has been demonstrated that NoV strains differed in their binding patterns and abilities to HBGAs and were associated with NoV susceptibility (Taylor et al., 2018).

Although GII NoVs show a higher prevalence in human and account for most of the NoV outbreaks globally (Kroneman et al., 2008), the prevalence of GI NoV had increased from 7.8% to 37.3% over the past decade (Hasing et al., 2013). For instance, GI NoV variants were widely detected in sub-Saharan Africa from 1993 to 2015 (Munjita, 2015). In southern China, GI.9, GI.2, and GI.3 were the most common GI NoV strains among asymptomatic carriers in coastal oyster farms (Wang et al., 2018). A surveillance in Taiwan during 2015–2019 revealed 16.5% NoV positive for GI, of which GI.3 was the most predominant genotype accounting for 36.8% and GI.2 accounting for 18.5%, thus suggesting close monitoring of GI NoVs (Chiu et al., 2020). The surveillance of NoV is challenging, since most infected individuals are asymptomatic or do not seek medical care. Hence, serological studies are required to determine the seroepidemiology and transmission dynamics of NoVs, which are impacted directly by the rate of neutralizing antibody formation and their persistence (Simmons et al., 2013; Malm et al., 2014). The persistence of herd immunity to NoVs varied in different research groups (Johnson et al., 1990; Sharma et al., 2017). Hence, community-based studies of specific NoV strains are necessary to determine the dynamics and persistence of neutralizing antibodies, since they provide crucial information for vaccine development strategies and are the target for the vaccination (van Loben Sels and Green, 2019). Therefore, our study established serological data of GI NoVs using blockade antibodies as substitute indicators of neutralizing antibodies in a Jidong community-based prospective cohort to investigate the seroprevalence, seroincidence, HBGA susceptibility, and persistence of herd immunity.

## 2 Materials and methods

### 2.1 Study design and participants

This was a prospective cohort study conducted among residents of Jidong Community in Caofeidian District, Tangshan City, in north China. A total of 10,043 participants aged 18 years and above were recruited in 2014, of whom 9,078 participants signed informed consent forms and completed the baseline data and the first bio-specimen collection.

**Figure 1:**
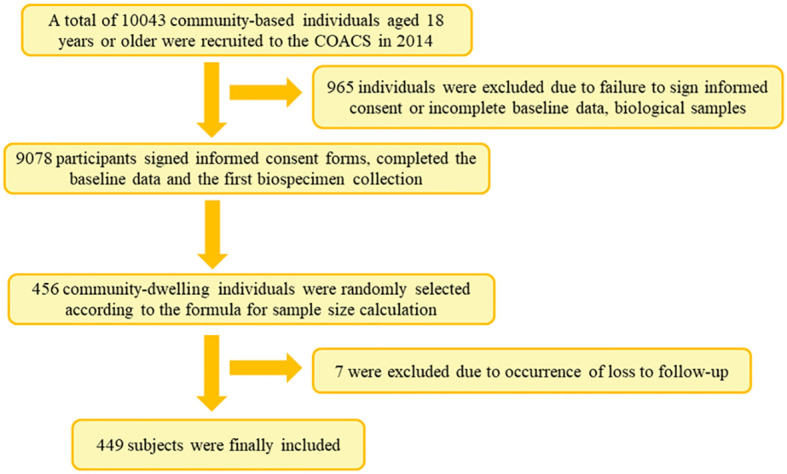
Flowchart of participants’ enrollment.

A preliminary experiment based on randomly selected 100 samples was conducted, and the seroprevalence rate of GI.2, GI.3, and GI.9 in participants was primarily determined to be 28.0%, 33.0%, and 29.0%, respectively. As a result, the minimal sample size of GI.2, GI.3, and GI.9 strains was respectively estimated to be 439, 347, and 418 using the following equation: [n = (Z_α_^2^)*P*(1 − *P*)/d^2^], in which *P* = 28.0%, 32.8%, and 29.2%, α = 0.05, Z_α_ = 1.96, and allowable error d = 0.15*P*. In the present study, 456 community residents were randomly selected for follow-up and biological sample collection until 2018. After excluding those lost in the follow-up, a representative sample of 449 individuals was included in the final analysis (**Figure 1**).

### 2.2 Data and biological sample collection

Demographic data were collected by completing a set of combined self-administered questionnaires. The collected variables were categorized as follows: marital status, educational status, and monthly income per capita (**Table 1**). Saliva samples were collected in 2016, while serum samples were collected annually from 2014 to 2018. All of the collected specimens were kept at -80°C for further experimental procedures.

**Table 1:** Demographic characteristics of the participants and seroprevalence against GI NoV in Jidong community-based cohort in 2014.

Demographic characteristics	Total(n=449)	GI.2		GI.3		GI.9	
		Seroprevalence % (95% CI)*^a^*	P value	Seroprevalence % (95% CI)	P value	Seroprevalence % (95% CI)	P value
Age group, years							
18-	132	16.7 (10.7-24.1)	0.845	33.3 (25.4-42.1)	0.551	22.0 (15.2-30.0)	0.588
30-	108	17.6 (10.9-26.1)		29.6 (21.2-39.2)		18.5 (11.7-27.1)	
40-	91	18.7 (11.3-28.2)		37.4 (27.4-48.1)		19.8 (12.2-29.4)	
50-	77	16.9 (9.3-27.1)		39.0 (28.0-50.8)		27.3 (17.7-38.6)	
60-75	41	24.4 (12.4-40.3)		41.5 (26.3-57.9)		26.8 (14.2-42.9)	
Sex							
Male	249	19.3 (14.6-24.7)	0.447	38.2 (32.1-44.5)	0.114	21.7 (16.7-27.3)	0.836
Female	200	16.5 (11.6-22.4)		31.0 (24.7-37.9)		22.5 (16.9-28.9)	
Ethnic group							
Han	436	18.6 (15.0-22.6)	0.086	35.1 (30.6-39.8)	0.747	22.2 (18.4-26.4)	0.556
ELSE	13	100 (75.3-100)		30.8 (9.1-61.4)		15.4 (1.9-45.4)	
Marital status							
Unmarried	38	23.7 (11.4-40.2)	0.449	44.7 (28.6-61.7)	0.358	18.4 (7.7-34.3)	0.419
Divorced/Widowed	12	8.3 (0.2-38.5)		41.7 (15.2-72.3)		8.3 (0.2-38.5)	
Married	399	17.8 (14.2-21.9)		33.8 (29.2-38.7)		22.8 (18.8-27.3)	
Education							
Illiteracy/primary school	13	15.4 (1.9-45.4)	0.966	46.2 (19.2-74.9)	0.231	38.5 (13.9-68.4)	0.350
Junior high school	167	18.0 (12.5-24.6)		38.9 (31.5-46.8)		21.6 (15.6-28.6)	
College or higher	269	18.2 (13.8-23.4)		32.0 (26.4-37.9)		21.6 (16.8-27.0)	
Income							
≤¥3,000	165	16.4 (11.1-22.9)	0.606	37.0 (29.6-44.8)	0.691	19.4 (13.7-26.3)	0.354
¥3,001–5,000	244	19.7 (14.9-25.2)		33.2 (27.3-39.5)		24.6 (19.3-30.5)	
>¥5000	40	15.0 (5.7-29.8)		37.5 (22.7-54.2)		17.5 (7.3-32.8)	

^a^95% CI: 95% confidence interval of the positive frequency.

### 2.3 Detection of HBGAs in saliva

The HBGA phenotypes of blood types (A, B, and O blood type), Lewis types (Le^a^, Le^b^, Le^x^, and Le^y^) of the saliva samples were determined by enzyme immunoassays (EIAs) using the corresponding monoclonal antibodies against individual HBGAs, as described earlier (Huang et al., 2005; Zhang et al., 2015). Briefly, boiled saliva samples at a dilution of 1:1,000 were coated on 96-well plates (Costar, Corning, NY, USA) overnight at 4°C. After blocking with 5% nonfat milk in phosphate buffered saline (PBS), the monoclonal antibodies specific for A (Z2A), B (Z5H-2), H (87-N) (Santa Cruz, CA, USA), Le^a^ (BG-5), Le^b^ (BG-6), Le^x^ (BG-7), and Le^y^ (BG-8) antigens (Signet Laboratories Inc., Dedham, MA, USA) were added. Then, horseradish peroxidase (HRP)-conjugated goat anti-mouse IgG or IgM (Abcam, UK; 1:3,000) was added, followed by signal development with 3,3’,5,5’-tetramethylbenzidine (TMB). The cutoff value was set at OD450 = 0.2, a value of the mean of the background/blank wells adding a triple standard deviation (Huang et al., 2005; Zhang et al., 2015). The control and experimental wells in the 96-well costar plates were arranged in parallel repeats. As a measure of quality control, well-defined positive and negative saliva samples were added to each plate.

### 2.4 HBGA blockade assays

Due to the lack of cell culture and small animal infection models, an HBGA-based blockade assay, showing the ability of serum antibody to prevent NoV proteins binding to HBGA, has been accepted as a surrogate for neutralization worldwide (Cannon et al., 2009; Reeck et al., 2010; Malm et al., 2014; Jin et al., 2016; Sharma et al., 2017). HBGA blockade assays were based on a saliva-based HBGA P protein-binding assay (Yang et al., 2010). Saliva samples that demonstrated strong binding signals were selected as described previously. Saliva from Volunteer 23 (V23, positive to phenotype A) was used for GI.2 and GI.3 NoV, and saliva from Volunteer 36 (V36, positive to phenotype O) was used for GI.9 NoV (Dai et al., 2017; Xie et al., 2020). In brief, saliva samples (1:1,000) were coated onto plates. Then, GI.2, GI.3, or GI.9 P proteins were added at the concentration of 0.2–0.5 µg/mL. Anti-GI.2, GI.3, and GI.9 in-house-made mouse sera (1:3,000) were then used to detect the bound P proteins. HRP-conjugated goat anti-mouse IgG (1:3,000) and TMB substrate kit were used to detect the binding of P proteins.

An extra step was performed for HBGA blockade assay. For the blocking effects, a preincubation of P protein with serum samples for 1 h at 37°C was added to the saliva-coated plates. P proteins without preincubation with serum samples, which demonstrated OD450 values approximately 0.7–1.3, were used as a positive control. The blocking index was calculated using the following equation: 100 – (OD for wells with serum/OD for wells without serum) × 100. Finally, OD450 values were measured for each sample, and those samples that showed at least 50% of blockade were identified as positive for blockade antibody (Cannon et al., 2009; Reeck et al., 2010; Xie et al., 2020). Well-defined positive and negative serum samples for blockade assay were also included in each plate.

### 2.5 Statistical analysis

The distribution characteristics of seroprevalence and seroincidence were revealed using Pearson chi-square and Fisher’s precision probability tests. The seroprevalence and seroincidence were estimated using the exact Clopper–Pearson with 95% confidence intervals (95% CIs) of MedCalc software. The person-years for seroincidence of GI NoV depended on the years contributed by those participants from serum antibody negative to positive. The associated factors with seroprevalence of GI NoV were determined using the generalized estimating equation (GEE), while correlations between HBGA phenotypes and different GI NoV blockade antibody were analyzed using logistic regression. Pairwise comparison was used for comparison between two groups. The duration persistence and kinetic of anti-GI NoV blocking antibodies were estimated by a generalized linear model (GLM) based on the observed data from 2014 to 2018. Differences in antibody decay rates were shown as empirical P-values, calculated by the Fisher’s permutation test in the Stata software. Statistical analyses were conducted with IBM SPSS software version 26.0 (SPSS Inc., Chicago, IL, USA). A P-value <0.05 (two-sided) was considered to be statistically significant.

## 3 Results

### 3.1 Baseline characteristics of study participants

A total of 449 participants were included in the final analysis, of whom 200 (44.5%) were women, with a mean age of 40.3 ± 12.7 years. Most were Han (97.1%) and married (88.9%); 269 participants (59.9%) having a degree or higher were educated. In addition, 244 (54.3%) earned a monthly income per capita of between 3,001 and 5,000 RMB (**Table 1**).

### 3.2 Seroprevalence of blockade antibodies against GI NoV

**Figure 2:**
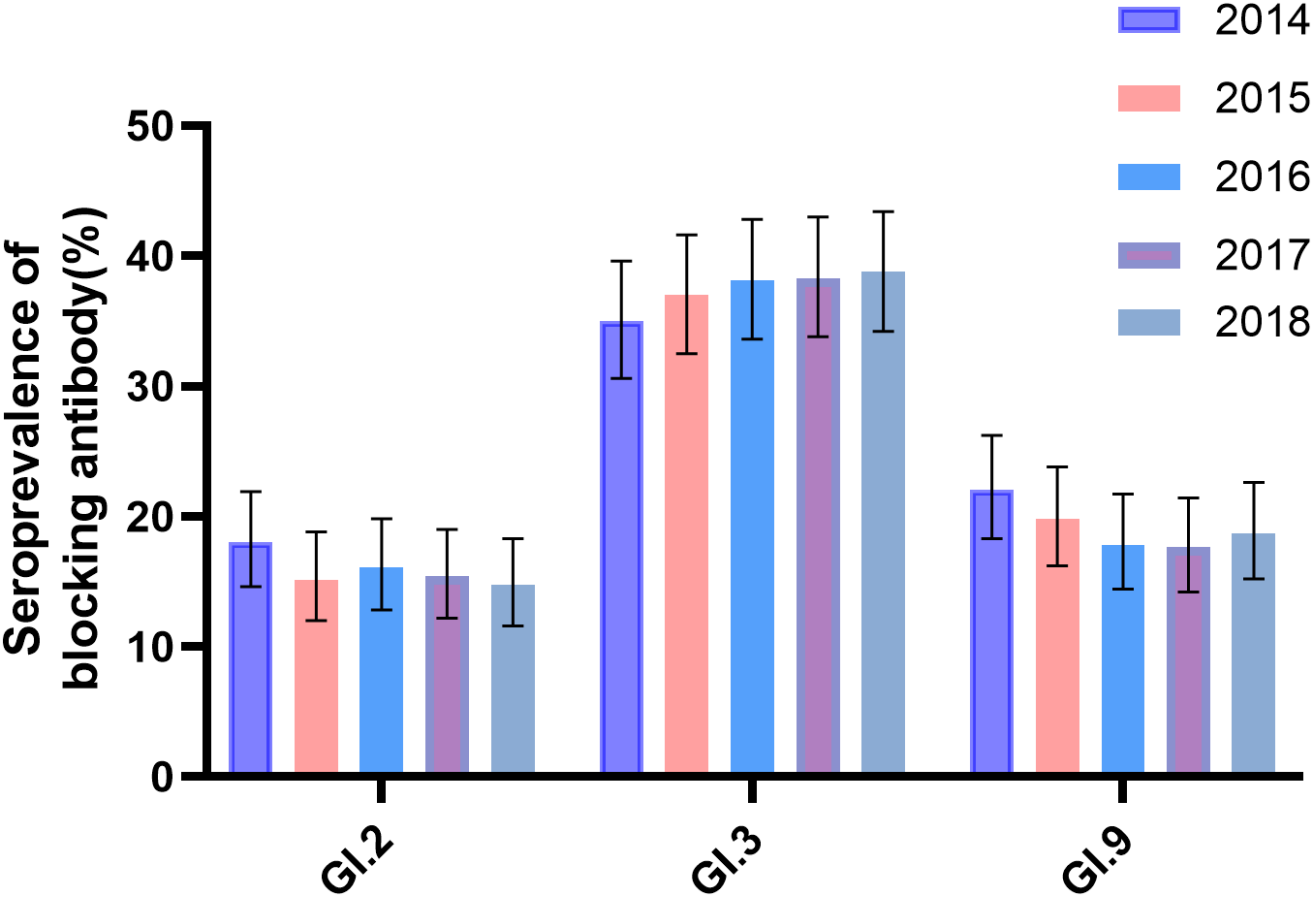
Seroprevalence of anti-GI blockade antibodies in 2014–2018. Anti-GI.2, GI.3, and GI.9 antibodies were tested annually in 449 participants. The y-axis indicated the seroprevalence. The colored bars indicated the corresponding seropositive rates for different years. Error bars indicated a 95% confidence interval.

The seroprevalence of GI.2 varied from 14.7% to 18.5% as follows from 2014 to 2018: 18.0% (81/449, 95% CI: 14.6%–21.9%), 15.1% (68/449, 95% CI: 12.0%–18.8%), 16.0% (72/449, 95% CI: 12.8%–19.8%), 15.4% (69/449, 95% CI: 12.2%–19.0%), and 14.7% (66/449, 95% CI: 11.6%–18.3%), with the highest in 2014 and lowest in 2018. In contrast, seroprevalence of GI.3 varied from 35.0% to 38.3% as follows: 35.0% (157/449, 95% CI: 30.6%–39.6%), 37.0% (166/449, 95% CI: 32.5%–41.6%), 38.1% (171/449, 95% CI: 33.6%–42.8%), 38.3% (172/449, 95% CI: 33.8%–43.0%), and 38.8% (174/449, 95% CI: 34.2%–43.4%), with the highest in 2018 and the lowest in 2014. For GI.9, it varied from 17.6% to 22.0% as follows: 22% (99/449, 95% CI: 18.3%–26.2%), 19.8% (89/449, 95% CI: 16.1%–23.8%), 17.8% (80/449, 95% CI: 14.4%–21.7%), 17.6% (79/449, 95% CI: 14.2%–21.4%), and 18.7% (84/449, 95% CI: 15.2%–22.6%), with the highest in 2014 and the lowest in 2017 (**Figure 2**). There was no significant variation in the seroprevalence of the three strains during the 5-year period (P > 0.05, **Figure 2**). The demographic characteristics of the seroprevalence of GI.2, GI.3, and GI.9 NoVs in the study population were not statistically significant at baseline in 2014 (P > 0.05, **Table 1**).

### 3.3 Factors associated with seroprevalence to GI.2, GI.3, and GI.9 NoV, 2014–2018

Individuals aged 50–59 years were significantly associated with the prevalence of GI.3 (OR = 1.757, P = 0.033) and GI.9 NoV (OR = 2.039, P = 0.022) during 2014–2018. In addition, individuals with married status were negatively associated with the prevalence of GI.3 (OR = 0.461, P = 0.002) as shown in **Table 2**.

**Table 2:** Factors associated with the prevalence of GI.2, GI.3, and GI.9 NoVs in Jidong community-based cohort, 2014–2018.

Demographic characteristics	GI.2		GI.3		GI.9	
	OR (95% CI)*^a^*	P value	OR (95% CI)	P value	OR (95% CI)	P value
Age group, years						
18-	1.000 (Ref.)		1.000 (Ref.)		1.000 (Ref.)	
30-	1.274 (0.787-2.063)	0.325	1.140 (0.744-1.746)	0.547	0.863 (0.501-1.487)	0.596
40-	1.441 (0.883-2.352)	0.144	1.316 (0.851-2.036)	0.217	1.260 (0.724-2.194)	0.414
50-	1.059 (0.602-1.863)	0.842	1.757 (1.047-2.948)	0.033^*^	2.039 (1.109-3.749)	0.022^*^
60-75	1.076 (0.568-2.040)	0.822	1.111 (0.606-2.037)	0.733	1.974 (0.915-4.256)	0.083
Sex						
Male	1.000 (Ref.)		1.000 (Ref.)		1.000 (Ref.)	
Female	1.088 (0.780-1.517)	0.620	0.798 (0.569-1.215)	0.302	0.797 (0.558-1.139)	0.213
Ethnic group						
Han	1.000 (Ref.)		1.000 (Ref.)		1.000 (Ref.)	
ELSE	0.609 (0.176-2.109)	0.434	1.348 (0.598-3.039)	0.472	1.193 (0.383-3.720)	0.760
Marital status						
Unmarried	1.000 (Ref.)		1.000 (Ref.)		1.000 (Ref.)	
Divorced/Widowed	0.447 (0.115-1.733)	0.244	1.046 (0.396-2.763)	0.928	0.942 (0.225-3.946)	0.935
Married	0.776 (0.383-1.573)	0.482	0.461 (0.284-0.750)	0.002^**^	1.547 (0.653-3.669)	0.322
Education						
Illiteracy/primary school	1.000 (Ref.)		1.000 (Ref.)		1.000 (Ref.)	
Junior high school	1.506 (0.409-5.543)	0.538	0.900 (0.345-2.344)	0.829	1.000 (0.324-3.088)	0.999
College or higher	1.671 (0.454-6.160)	0.440	0.773 (0.286-2.092)	0.613	1.185 (0.365-3.854)	0.777
Income						
≤¥3,000	1.000 (Ref.)		1.000(Ref.)		1.000 (Ref.)	
¥3,001–5,000	0.903 (0.606-1.348)	0.619	1.054 (0.757-1.468)	0.756	1.205 (0.773-1.879)	0.410
>¥5000	0.536 (0.299-0.962)	0.037	0.816 (0.468-1.424)	0.475	0.894 (0.429-1.861)	0.765

^a^OR, odds ratio; 95% CI, 95% confidence interval.

*P < 0.05, **P < 0.01. The bold values indicate statistical significance.

### 3.4 Factors associated with the seroincidence of GI.2, GI.3, and GI.9 NoV, 2014–2018

During the 4-year follow-up, 1,506, 1,130, and 1,449 person-years for participants with initial antibody negative were followed, and a total of 149, 241, and 152 participants showed NoV seroconversion for GI.2, GI.3, and GI.9 NoV, respectively. Thus, the seroincidences of the studied GI NoVs were 10 (95% CI: 8–12), 21 (95% CI: 19–24), and 11 (95% CI: 9–12) per 100 person-years for GI.2, GI.3, and GI.9 NoV between 2014 and 2018, respectively. Each observational year showed a stable seroincidence over the period (**Table 3**).

**Table 3:** Factors associated with seroincidence of GI.2, GI.3, and GI.9 NoV in Jidong community-based cohort, 2014–2018.

Demographic characteristics	GI.2			GI.3			GI.9		
	Person-years followed	Seroincidence *^a^* (per 100 person-years) (95% CI)*^b^*	P value	Person-years followed	Seroincidence (per 100 person-years) (95% CI)	P value	Person-years followed	Seroincidence (per 100 person-years)(95% CI)	P value
Age group, years									
18-	257	8 (5-12)	0.487	197	17 (12-23)	0.443	258	5 (2-8)	0.012^*^
30-	494	10 (8-13)		385	20 (16-24)		488	7 (5-10)	
40-	300	12 (9-16)		235	19 (14-24)		301	11 (8-15)	
50-	224	10 (6-15)		150	23 (17-31)		210	12 (8-18)	
60-80*^c^*	231	8 (5-13)		163	23 (17-31)		192	8 (5-14)	
Total*^d^*	1506	10 (8-12)		1130	21 (19-24)		1449	11 (9-12)	
Sex									
Male	833	9 (7-11)	0.082	589	22 (19-27)	0.059	791	9 (7-11)	0.777
Female	673	11 (9-14)		541	19 (16-23)		658	8 (6-11)	
Ethnic group									
Han	1460	10 (9-12)	1.000	1100	20 (18-23)	0.369	1407	9 (7-10)	0.574
ELSE	46	9 (2-21)		30	27 (12-46)		42	5 (1-16)	
Marital status									
Unmarried	125	8 (4-14)	0.772	81	37 (27-49)	<0.001^***^	133	5 (2-10)	0.145
Divorced/Widowed	43	9(3-22)		21	33 (15-57)		41	12 (4-26)	
Married	1338	10 (9-12)		1028	19 (17-22)		1275	9 (7-11)	
**Education**						0.454			
Illiteracy/primary school	46	4 (0.5-15)	0.509	29	14 (4-32)		38	3 (0-14)	0.515
Junior high school	565	10 (8-13)		398	22 (18-27)		523	9 (6-11)	
College or higher	895	10 (8-12)		703	20 (17-23)		881	9 (7-11)	
Income									
≤¥3,000	552	9 (7-12)	0.744	401	19 (16-23)	0.263	533	7 (5-10)	0.277
¥3,001–5,000	813	10 (7-11)		624	22 (19-26)		782	10 (8-12)	
>¥5000	141	9 (8-13)		105	16 (10-25)		134	8 (4-13)	
Follow-up year									
2015	368	8 (5-11)	0.434	292	20 (15-25)	0.623	350	9 (6-12)	0.676
2016	381	11 (8-15)		283	21 (16-26)		360	8 (6-11)	
2017	377	10 (7-13)		278	19 (14-24)		369	7 (5-11)	
2018	380	11 (8-14)		227	23 (18-29)		370	10 (7-13)	

^a^The new infection of GI NoV was defined as the seroconversion from negative to positive for the corresponding genotype of NoV. The seroincidence of GI NoV was determined by dividing the number of newly infected individuals by the person-years observed during the study period from 2014 to 2018.

^b^95% CI: 95% confidence interval.

^c^Person-years observed in the 60–80-year age group resulted from the follow-up of participants aged 60–75 years old.

^d^The total number of participants with seroconversion for GI.2, GI.3, and GI.9 was 149, 241, and 152, respectively.

*P < 0.05, ***P < 0.001. The bold values indicate statistical significance.

A significant difference was found in seroincidence of GI.9 when all age groups were compared (P = 0.012, **Table 3**). It was relatively low at 5.0/100.0 person-years in those aged 18–29 years but gradually increased to reach a peak of 12.0/100.0 person-years in the age group 50–59 years, which later declined to 8.0/100.0 person-years in those aged 60–80 years (**Table 3**). The seroincidence of GI.2 and GI.3 also exhibited similar age trends but without statistical significance. The seroincidence of GI.3 was significantly low in the married population compared to the unmarried (19.0/100.0 person-years vs. 37.0/100.0 person-years, P < 0.001). However, the seroincidence of GI.2 exhibited no statistical significance with any of the sociodemographic variables, as illustrated in **Table 3**.

### 3.5 Demographic distribution between GI NoV antibody positive and negative groups

Among 449 participants, 196 (43.7%), 302 (67.3%), and 182 (40.5%) respectively tested at least once positive to GI.2, GI.3, or GI.9 during 2014–2018. There was a significant difference between marital status for GI.3 NoV between GI NoV antibody positive and negative groups. No other significant difference of demographic distribution was observed (**Table 4**).

**Table 4:** Demographic distribution between antibody positive and negative groups to GI NoV in Jidong community-based cohort, 2014–2018.

Demographic characteristics		Total (n=449)	GI.2			GI.3			GI.9		
			No. (%) of positive (n=196)	No. (%) of negative (n=253)	P value	No. (%) of positive (n=302)	No. (%) of negative (n=147)	P value	No. (%) of positive (n=182)	No. (%) of negative (n=269)	P value
Age group, years											
18-		132 (29.4)	54 (27.6)	78 (30.8)	0.651	91 (30.1)	41 (27.9)	0.543	47 (25.8)	85 (31.8)	0.136
30-		108 (24.1)	49 (25.0)	59 (23.3)		66 (21.9)	42 (28.6)		37 (20.3)	71 (26.6)	
40-		91 (20.3)	45 (23.0)	46 (18.2)		61 (20.2)	30 (20.4)		42 (23.1)	49 (18.4)	
50-		77 (17.1)	30 (15.3)	47 (18.6)		56 (18.5)	21 (14.3)		35 (19.2)	42 (15.7)	
60-75		41 (9.1)	18 (9.2)	23 (9.1)		28 (9.3)	13 (8.8)		21 (11.5)	20 (7.5)	
Total		449	196 (43.7)	253 (56.35)		302 (67.2)	147 (32.7)		182 (40.5)	267 (59.5)	
Sex											
Male		249 (55.5)	108 (55.1)	141 (55.7)	0.894	176 (58.3)	73 (49.7)	0.085	102 (56.0)	147 (55.1)	0.836
Female		200 (44.5)	88 (44.9)	112 (44.3)		126 (41.7)	74 (50.3)		80 (44.0)	120 (44.9)	
Ethnic group											
Han		436 (97.1)	192 (98.0)	244 (96.4)	0.342	292 (96.7)	144 (98.0)	0.451	178 (97.8)	258 (96.6)	0.467
ELSE		13 (2.9)	4 (2.0)	9 (3.6)		10 (3.3)	3 (2.0)		4 (2.2)	9 (3.4)	
Marital status											
Unmarried		38 (8.5)	14 (7.1)	24 (9.5)	0.500	33 (10.9)	5 (3.4)	0.011^*^	10 (5.5)	28 (10.5)	0.175
Divorced/Widowed		12 (2.7)	4 (2.0)	8 (3.2)		10 (3.3)	2 (1.4)		5 (2.7)	7 (2.6)	
Married		399 (88.9)	178 (90.8)	221 (87.4)		259 (85.8)	140 (95.2)		167 (91.8)	232 (86.9)	
Education											
Illiteracy/primary school		13 (2.9)	3 (1.5)	10 (4.0)	0.316	9 (3.0)	4 (2.7)	0.975	6 (3.3)	7 (2.6)	0.910
Junior high school		167 (37.2)	74 (37.8)	93 (36.8)		113 (37.4)	54 (36.7)		68 (37.4)	99 (37.1)	
College or higher		269 (59.9)	119 (60.7)	150 (59.3)		180 (59.6)	89 (60.5)		108 (59.3)	161 (60.3)	
Income											
≤¥3,000		165 (36.7)	70 (35.7)	95 (37.5)	0.771	109 (36.1)	56 (38.1)	0.470	62 (34.1)	103 (38.6)	0.501
¥3,001–5,000		244 (54.3)	110 (56.1)	134 (53.0)		169 (56.0)	75 (51.0)		105 (57.7)	139 (52.1)	
>¥5000		40 (8.9)	16 (8.2)	24 (9.5)		24 (7.9)	16 (10.9)		15 (8.2)	25 (9.4)	

*P < 0.05. The bold values indicate statistical significance.

### 3.6 Distributions of HBGA phenotypes between antibody positive and negative groups to GI NoV

The distribution of HBGA phenotypes in the participants was as follows: phenotype A, 31.2%; phenotype B, 18.0%; phenotype AB, 19.4%; phenotype O^+^ (group O with Rh antigen positive), 18.5%; and 12.9% for O^-^ phenotype (group O with Rh antigen negative). In terms of the Lewis phenotype, 12.9% of the individuals were Le^a+/^Le^x+^, 56.1% were Le^b+/^Le^y+^, and 31.0% were Le^a+b+^/Le^x+y+^. Majority of the participants (87.1%, 391/449) were classified as secretors (**Table 5**).

**Table 5:** Distributions of HBGA phenotypes between antibody positive and negative groups to GI NoV in Jidong community-based cohort, 2014–2018.

HBGAs	Total (n=449)	GI.2			GI.3			GI.9		
		No. (%) of positive (n=196)	No. (%) of negative (n=253)	P value	No. (%) of positive (n=302)	No. (%) of negative (n=147)	P value	No. (%) of positive (n=182)	No. (%) of negative (n=269)	P value
ABO blood types										
A	140 (31.2)	61 (31.1)	79 (31.2)	0.001^**^	103 (34.1)	37 (25.2)	<0.001^***^	58 (31.9)	82 (30.7)	0.245
B	81 (18.0)	46 (23.5)	35 (13.8)		45 (14.9)	36 (24.5)		25 (13.7)	56 (21.0)	
AB	87 (19.4)	32 (16.3)	55 (21.7)		52 (17.2)	35 (23.8)		34 (18.7)	53 (19.9)	
O**^+^**	83 (18.5)	43 (21.9)	40 (15.8)		73 (24.2)	10 (6.8)		40 (22.0)	43 (16.1)	
O**^-^**	58 (12.9)	14 (7.1)	44 (17.4)		29 (9.6)	29 (19.7)		25 (13.7)	33 (12.4)	
Lewis phenotypes										
Le^a+^/Le^x+^	58 (12.9)	14 (7.1)	44 (17.4)	0.005^**^	29 (9.6)	29 (19.7)	0.010^*^	25 (13.7)	33 (12.4)	0.760
Le^b+^/Le^y+^	252 (56.1)	115 (58.7)	137 (54.2)		174 (57.6)	78 (53.1)		104 (57.1)	148 (55.4)	
Le^a+b+^/Le^x+y+^	139 (31.0)	67 (34.2)	72 (28.5)		99 (32.8)	40 (27.2)		53 (29.1)	86 (32.2)	
Secretor status										
Nonsecretor	58 (12.9)	14 (7.1)	44 (17.4)	0.001^**^	29 (9.6)	29 (19.7)	0.003^**^	25 (13.7)	33 (12.4)	0.669
Secretor	391 (87.1)	182 (92.9)	209 (82.6)		273 (90.4)	118 (80.3)		157 (86.3)	234 (87.6)	

*P < 0.05, **P < 0.01, ***P < 0.001. The bold values indicate statistical significance.

Compared to the group with antibody negative to GI.2 NoV, the positive group had a significantly larger proportion of A phenotype (31.1%) but a lower proportion of O^-^ phenotype (7.1%; P = 0.001), and similar results were observed for GI.3 NoV (P < 0.001, **Table 5**). For antibody-positive group to GI.2 and GI.3 NoV, a higher proportion of Le^b+^/Le^y+^ (P_GI.2_ = 0.005, P_GI.3_ = 0.010) and a lower proportion of nonsecretor (P_GI.2_ = 0.001, P_GI.3_ = 0.003) were observed, indicating the individuals with Le^a+^/Le^x+^ or the nonsecretor less susceptible to GI.2 or GI.3 NoV (**Table 5**). However, no statistical difference of HBGA distribution was found across groups for GI.9 (P = 0.245, **Table 5**).

### 3.7 Positive rates of blockade antibody for participants with different HBGA phenotypes

**Figure 3:**
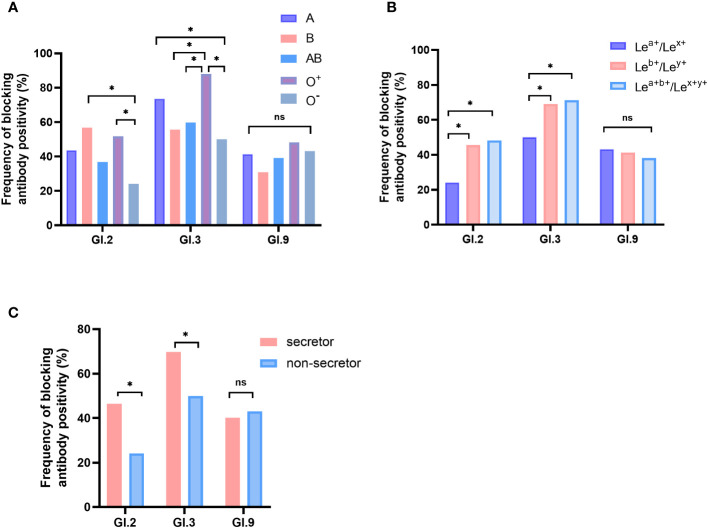
Blockade antibody positive rates of GI NoVs for participants with different ABO blood types **(A)**, Lewis phenotypes **(B)**, and secretor status **(C)**. The y-axis indicated the frequency of blockade antibodies. Different colored bars showed different human histo-blood group antigen (HBGA) phenotypes. *P < 0.05; NS, nonsignificant.

Significant differences were observed in positive rates of blockade antibody among participants with different HBGA phenotypes for GI.2 and GI.3, but not for GI.9 NoV. For GI.2 NoV, the positive rate of blockade antibody of individuals with O^-^ phenotype was significantly lower than that of individuals with B (24.1% vs. 56.8%, P < 0.05) and O^+^ phenotype (24.1% vs. 51.8%, P < 0.05). For GI.3 NoV, blockade antibody positive rate of individuals with O^+^ phenotype was the highest (88.0%) and followed with those with A (73.6%), AB (59.8%), B (55.6%), and O^-^ phenotype (50.0%) with significant differences (**Figure 3A**). Positive rates were significantly higher for both individuals with Le^b+^/Le^y+^ (GI.2: 45.6% and GI.3: 69.0%) and Le^a+b+^/Le^x+y+^ (GI.2: 48.2%, GI.3: 71.2%) phenotypes than that for the Le^a+^/Le^x+^ phenotype (GI.2: 24.1%, GI.3: 50.0%, **Figure 3B**), and similar findings were observed for secretor status (**Figure 3C**).

### 3.8 Association of HBGAs with antibodies against GI NoV strains

Compared to the O^-^ phenotype, participants with A and O^+^ for GI.2 and GI.3 NoV and phenotype B for GI.2 NoV showed elevated susceptibility. Infection risk of GI.2 and GI.3 strains was also significantly higher among those with Le^b+^/Le^y+^ (GI.2: OR = 2.843; GI.3: OR = 2.363) and those with Le^a+b+^/Le^x+y+^ (GI.2: OR = 3.139; GI.3: OR = 2.371). Secretors had a significantly higher risk of infection with GI.2 (OR = 2.950) and GI.3 (OR = 2.366) NoV than that of the nonsecretors. However, no significant association susceptibility to GI.9 strain was observed among individuals with different HBGA phenotypes (**Table 6**).

**Table 6:** Association of HBGAs with antibodies against GI NoV strains using multivariate logistic analysis.

HBGAs	GI.2		GI.3		GI.9	
	OR(95% CI)*^a^*	Adj-OR(95% CI)*^b^*	OR(95% CI)	Adj-OR(95% CI)	OR(95% CI)	Adj-OR(95% CI)
ABOblood type						
A	**2.427** (1.220-4.828)^*^	**2.541** (1.265-5.106)^**^	**2.784** (1.472-5.265)^**^	**2.847** (1.472-5.506) ^**^	0.934 (0.503-1.734)	1.015 (0.539-1.911)
B	**4.131** (1.961-8.701)^***^	**4.441** (2.084-9.464)^***^	1.094 (0.636-2.458)	1.317 (0.653-2.658)	0.589 (0.292-1.189)	0.662 (0.324-1.353)
AB	1.829 (0.870-3.843)	1.968 (0.921-4.204)	1.300 (0.760-2.903)	1.397 (0.692-2.819)	0.847 (0.431-1.663)	0.960 (0.479-1.925)
O**^+^**	**3.379** (1.613-7.079)^**^	**3.749** (1.761-7.981)^**^	**7.300** (3.159-16.870)^***^	**8.065** (3.393-19.173) ^***^	1.228 (0.625-2.411)	1.442 (0.719-2.890)
O**^-^**	1.000 (Ref.)	1.000(Ref.)	1.000(Ref.)	1.000(Ref.)	1.000(Ref.)	1.000(Ref.)
Lewis phenotypes						
Le^b+^/Le^y+^	**2.638** (1.377- 5.056)^**^	**2.843** (1.463-5.522)^**^	**2.231** (1.249-3.984)^**^	**2.363** (1.290-4.330)^**^	0.928 (0.521-1.652)	1.088 (0.601-1.970)
Le^a+b+^/Le^x+y+^	**2.925** (1.471-5.815)^**^	**3.139** (1.562-6.306)^**^	**2.475** (1.315-4.658)^**^	**2.371** (1.238-4.542)^**^	0.813 (0.437-1.516)	0.846 (0.448-1.599)
Le^a+^/Le^x+^	1.000(Ref.)	1.000(Ref.)	1.000(Ref.)	1.000(Ref.)	1.000(Ref.)	1.000(Ref.)
Secretor status						
Secretor	**2.737** (1.453-5.156)^**^	**2.950** (1.549-5.619)^**^	**2.314** (1.324-4.043)^**^	**2.366** (1.326-4.222)^**^	0.886 (0.507-1.547)	0.991 (0.560-1.754)
Nonsecretor	1.000(Ref.)	1.000(Ref.)	1.000(Ref.)	1.000(Ref.)	1.000(Ref.)	1.000(Ref.)

^a^OR, odds ratio; 95% CI, 95% confidence interval.

^b^Adj OR: adjusted odds ratio, logistic regression model adjusted for age, sex, ethnicity, marital status, education level, and monthly income per capita of household obtained.

^c^*P < 0.05, ** P < 0.001, *** P < 0.001. The bold values indicate statistical significance.

### 3.9 Dynamic and duration analysis of blockade antibody against GI.2, GI.3, and GI.9 NoV

**Figure 4:**
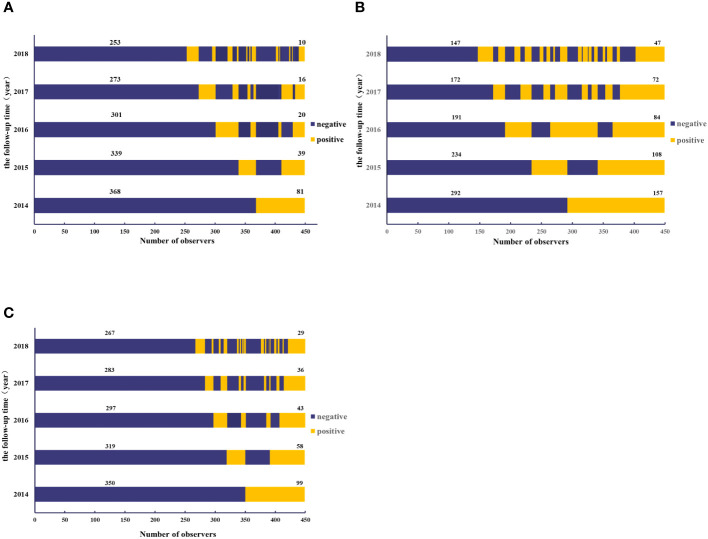
Dynamics of blockade antibody against GI.2 **(A)**, GI.3 **(B)**, and GI.9 **(C)** NoV from 2014 to 2018. The y-axis indicated the follow-up year, and the x-axis illustrated the number of observations. Blue indicated the number of participants with antibody positive, whereas yellow indicated those with antibody negative. The number above the blue bar indicated individuals with antibody persistent positive, and the number above the yellow bar indicated individuals with antibody persistent negative.

In the initial antibody positive population, repeated seroconversion (initially positive to negative and turned positive again) during follow-up was defined as reinfection. A total of 81, 157, and 99 individuals were respectively found to be seropositive of anti-GI.2, GI.3, and GI.9 NoV blockade antibodies in 2014. Among them, 66, 104, and 72 entered the anti-GI.2, GI.3, and GI.9 NoV blockade antibody persistence and seronegative conversion rate analysis, respectively, and the remaining 15, 53, and 27 participants with sero-reinfection were excluded to avoid reinfection affecting the accuracy of the results. During the follow-up period, 5-year persistent positive rates of 29.9% (47/157), 29.3% (29/99), and 12.3% (10/81) against GI.3, GI.9, and GI.2 NoV were observed (**Figure 4**).

The overall negative conversion rates for GI.2, GI.3, and GI.9 strains of blockade antibodies in the initially seropositive population were 84.8% (55/66), 54.8% (57/104), and 59.7% (43/72), respectively. Most of the antibody reversal occurred within the first year after seropositivity, with anti-GI.2 and GI.9 NoV antibodies having the highest serum reversal rate of 50% (33/66) and 34.7% (25/72) after 1 year in 2015. However, anti-GI.3 NoV antibodies had the highest negative conversion rates of 34.7% (25/72) in the fourth year. The three strains exhibited statistically significant differences in serum reversal rates across years (**Supplementary Table S1**).

**Figure 5:**
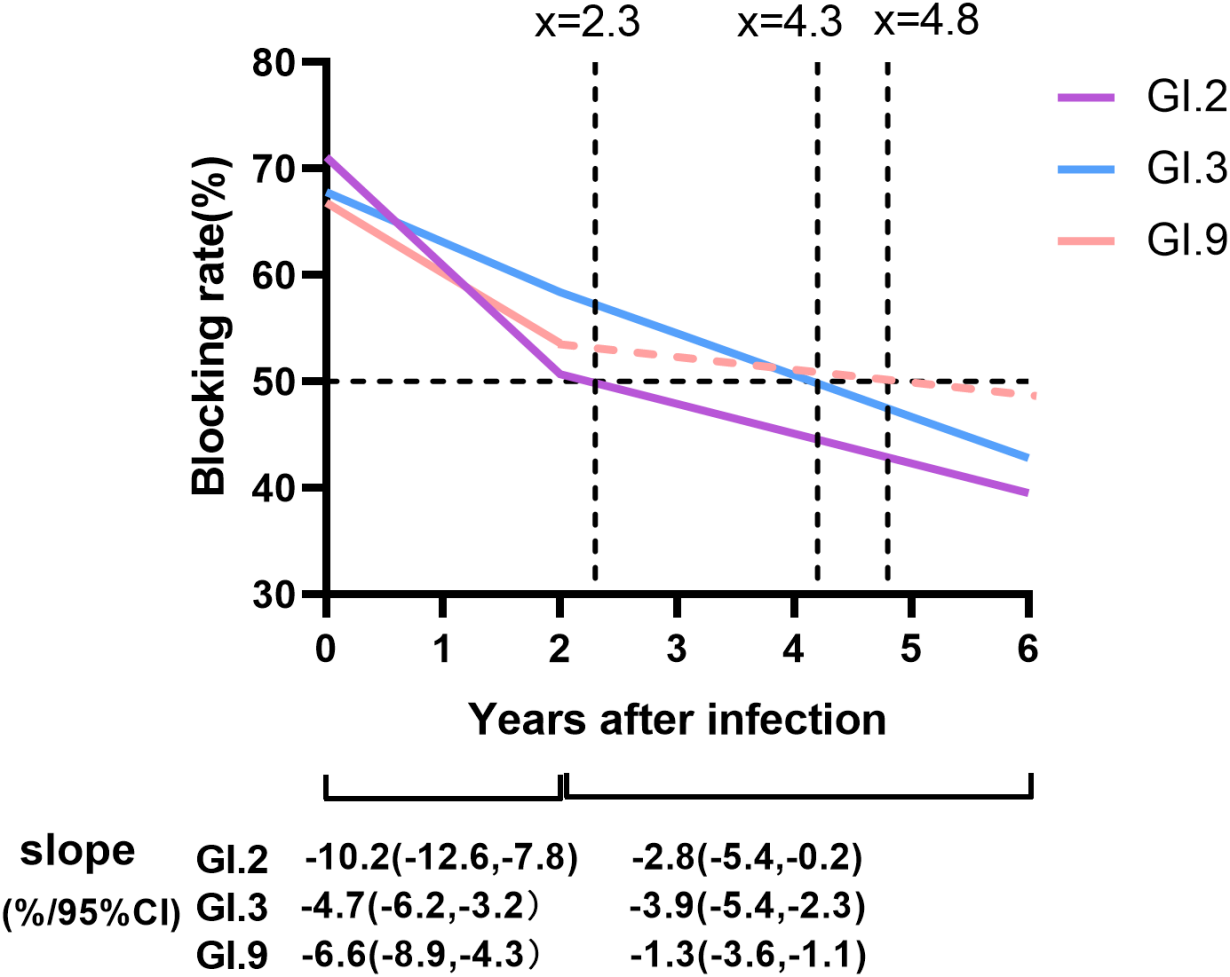
Duration and dynamic of protective immunity against GI NoV. Colored lines represented the duration and dynamic of blockade antibody estimated by generalized linear model (GLM) model analysis, in which the solid lines referred to the dynamic of antibody with statistical significance, while dash lines without statistical significance. Dot lines indicated the cutoff values of the anti-GI NoV antibody (blocking rate = 50%). Slopes were estimated by GLM models.

The blockade rates of GI NoVs antibodies showed an overall decreasing trend from year to year with an overall mean decay rate of -5.9%/year, -3.6%/year, and -4.0%/year for GI.2, GI.3, and GI.9, respectively. Notably, the antibodies of GI.2 and GI.9 strains both declined more rapidly in the first 1–2 years after positivity, with decay rates of -10.2%/year (95% CI: -12.6%/year, -7.8%/year) for GI.2 and -6.6%/year (95% CI: -8.9%/year, -4.3%/year) for GI.9 with a statistically significant trend (P < 0.001, **Figure 5**). However, for the GI.3 strain, the decay rate of the blocking effect after infection in the first 2 years was similar to that second 2 years, at -4.7%/year and -3.9%/year (95% CI: -5.4%/year, -2.3%/year). The least persistent antibody among the three GI NoV strains was estimated to be GI.2 at approximately 2.3 years, while anti-GI.3 and GI.9 NoV antibodies lasted for approximately 4.2 and 4.8 years, respectively (**Figure 5**).

## 4 Discussion

The present study reports the serological data on herd immunity and the persistence of blockade antibodies against GI NoVs in a Jidong community-based prospective cohort, 2014–2018. To the best of our knowledge, this is the first longitudinal study about the seroepidemiology of GI NoV using blockade antibody in the general natural population.

Seroepidemiological analyses have the significant advantage of reflecting current and past NoV infections (Liu et al., 2015; Thorne et al., 2018). Most seroepidemiologic studies of NoV have been based mainly on specific IgG antibodies, which are detected directly using NoV VLPs or P protein and always reflect cross-reactive antibodies left in the body from prior infection of a particular genotype, whose strong intragenomic cross-reactivity makes it impossible to distinguish between previous infections of different genotypes precisely (Parra and Green, 2014; Karangwa et al., 2017; Parra et al., 2017; Xie et al., 2020). However, the level of blockade antibodies as a surrogate of neutralizing antibody can suggest its ability to block the binding of NoV to HBGAs, which is conformation- and genotype-dependent (Huo et al., 2016). It has been proven to be correlated with the protection of NoV infection. In addition, little cross-reactivity of blockade antibody between genotypes of different immunophenotypes has been reported in our previous studies and other groups (Parra and Green, 2014; Huo et al., 2016; Dai et al., 2017; Karangwa et al., 2017; Xie et al., 2020). For example, our previous study on characterizing antigenic relatedness among GI NoVs revealed no statistical difference for the specific IgG titer, seroconversion rate, and increased folds among GI.2, GI.3, and GI.9 in IgG antibody in humans following GI.3 NoV infections while having strong blockade in homologous strain (GI.3), moderate intraimmunotype (GI.9) blockade, but weak interimmunotype (GI.2) blockade in blocking antibody (Xie et al., 2020). Therefore, the seroprevalence and seroincidence obtained based on blockade antibody assays are more indicative of the actual immune status of NoV in the population.

Our study found that the average seropositive rates for GI.2, GI.3, and GI.9 in Jidong community were 15.86%, 37.44%, and 19.18% from 2014 to 2018, without significant variation across years for each strain, suggesting that previous infections of GI NoV were stable and common in Jidong community. Seroincidences of GI.2, GI.3, and GI.9 were 10.0, 21.0, and 11.0 per 100 person-years, which were higher than the incidence of GI and GII NoVs recorded in the Chinese population based on stool samples (6/100 person-years) (Zhou et al., 2017). It is also higher than the incidence of NoV gastrointestinal disease in other countries, suggesting that the current incidence and disease burden of NoV may be underestimated (Phillips et al., 2010; Scallan et al., 2011; Tam et al., 2012; Verhoef et al., 2013). The underestimation may be due to the self-limiting nature of NoVs, leading to a large proportion of the population not seeking medical treatment and testing stool samples. As a result, the seroprevalence and underestimated incidence suggest enhancing and refining surveillance for GI NoV and fully demonstrating the necessity of implementing effective vaccine development.

Previous studies found that people with NoV AGE had two peaks of age, with the first peak at 6–23 months and a second peak after 40 years (Grytdal et al., 2016; Zhou et al., 2017). In the present study, individuals aged 50–59 years have higher seropositivity and seroincidence of GI.3 and GI.9, implying that age might play a role in infection with these strains. We also observed that the seroprevalence, seroincidence, and positive rate for different marriage statuses showed a significant difference with P values at 0.033, <0.001, and 0.011 for GI.3 NoV, which indicated that marriage statuses might be associated with GI.3 NoV infection. For GI.9 NoV, only seroprevalence for different marriage statuses showed a significant difference at 0.022, which required more evidence to clarify the association. The binding characteristics of NoV to HBGAs play an important role in host susceptibility, range of infection, and the prevalence and evolution of NoV (Chassaing et al., 2020). Consistent with the results of our previous findings (Xie et al., 2020) and the other group (Tan and Jiang, 2008) on the binding characteristics of GI NoV, phenotypes A, B (excluding GI.3), and O^+^ of ABO blood types, Le^b+^/Le^y+^ and Le^a+b+^/Le^x+y+^ of Lewis types, and secretor status were susceptibility factors for GI.2 and GI.3 NoV. However, HBGA status had no contribution to GI.9 antibody positivity and relative risk of infection, which might be due to broad-spectrum binding properties of GI.9 NoV to all HBGA phenotypes as described previously (Xie et al., 2020).

The dose of virus given to volunteers in all classic challenge studies was several thousand-fold greater than the small amount capable of causing human illness estimated to be 18–1,000 virus (Teunis et al., 2008). Teunis et al. (2008) demonstrated that immunity elicited by lower doses of NoV was more significant and robust than artificially stimulated doses in volunteer challenge studies, indicating that protective immunity generated by natural infections in the community may be more durable. A recent study showed that the aggregation of viruses in the stool stock used to prepare administered doses generally led to a suppressed dose response, such as fewer infections at lower doses and a higher infectious dose (ID_50_) and estimated the ID_50_ and diarrhea dose (DD_50_) to be between 2,400 and 3,400 RNA copies, and 21,000 and 38,000 RNA copies, respectively (Ramesh et al., 2020). Our study showed a high negative seroconversion rate (all exceeding 50.0%) of the anti-GI NoV antibodies during the 4 years of follow-up, especially for the GI.2 strain, which had an overall negative seroconversion rate of 84.8%. For the GI.2 and GI.9 incident samples, we observed a rapid reduction in the blocking effect of antibodies with serum reversal of antibodies in more than half of individuals within the first year of post-positivity. Interestingly, initial GI.3 strain-positive subjects had the highest rate of negative seroconversion (34.7%) in the fourth year, suggesting that GI.3 antibodies might confer more sustained protective immunity. This study estimated that anti-GI.3 and GI.9 NoV antibodies lasted approximately 4.2 and 4.8 years, respectively, maintaining a longer protective immunity. Our findings are consistent with a study that used a mathematical model to estimate NoV immunity to be 4.1–8.7 years (Simmons et al., 2013). Uncertainty on the duration of protective immunity is one of the critical challenges to vaccine development and application of immunization programs and the evaluation of vaccine efficacy in NoVs (Hallowell et al., 2019). The short duration of protection (e.g., ≤1 year) implies that multiple injections are required, resulting in a high cost of reagents. Additionally, the inconvenience caused by frequent injections might reduce individual willingness to be vaccinated. However, we found that protective immunity against all GI NoVs lasted for more than 2 years. In fact, GI.3 and GI.9 NoV lasted for more than 4 years, indicating cost-effectiveness and health benefits by vaccination. This finding is significantly higher than the previously estimated protective duration, providing a solid supporting basis for vaccine development and immunization programs.

The present study had several potential limitations. First, the study excluded those below the age of 18 years and thus could not provide actual data on under-5 children who are notably vulnerable. Second, seroepidemiological studies were only carried out at qualitative and semiquantitative levels. Further titer analysis of antibodies would be necessary to determine more accurate levels of herd immunity to NoV. Third, the study was unique in that it analyzed data from participants who were all from Jidong community in north China; hence, the findings should be interpreted with caution in relation to other regions. Fourth, there was only a small number of unmarried participants in comparison to married participants, thus further evidence is needed to give solid conclusion on GI NoV infection and marriage status. Fifth, the study did not collect data on symptoms and could not provide estimation on rates of asymptomatic infection. Finally, NoV GII is more prevalent worldwide, while this study focused on NoV GI seroepidemiology. Seroepidemiology of NoV GII is highly recommended to be conducted and is being performed by our group.

## 5 Conclusion

Seroprevalence of GI NoV in people over 18 years in the Jidong community was high, suggesting that previous infection of GI NoV was common. HBGA phenotypes were associated with GI.2 and GI.3 strain infections. The blockade antibodies produced by GI.2, GI.3, and GI.9 decreased at a certain rate over 5 years, lasting approximately 2.3, 4.2, and 4.8 years, respectively. Enhanced community surveillance and prevention and control of GI NoV are thus needed, especially the development and promotion of vaccines.

## Data availability statement

The original contributions presented in the study are included in the article/**Supplementary Material**. Further inquiries can be directed to the corresponding authors.

## Ethics statement

Ethical approval was obtained from Jidong oil-field of China National Petroleum Corporation and Nanfang Hospital of Southern Medical University. Additionally, all study subjects consented prior to enrollment in the study. The studies were conducted in accordance with the local legislation and institutional requirements. The participants provided their written informed consent to participate in this study. Written informed consent was obtained from the individual(s) for the publication of any potentially identifiable images or data included in this article.

## Author contributions

J-RY: Data curation, Formal analysis, Writing – original draft, Writing – review & editing. D-JX: Conceptualization, Investigation, Methodology, Writing – original draft, Writing – review & editing. J-HL: Data curation, Formal analysis, Writing – review & editing, Writing – original draft. MK: Writing – review & editing. LW: Formal Analysis, Writing – review & editing. YW: Formal analysis, Writing – review & editing. D-NJ: Formal analysis, Writing – review & editing. J-YX: Formal analysis, Writing – review & editing. J-XY: Formal analysis, Writing – review & editing. H-SD: Formal analysis, Writing – review & editing. F-YZ: Formal analysis, Writing – review & editing. Z-YL: Formal analysis, Writing – review & editing. X-FZ: Data curation, Project administration, Resources, Writing – review & editing. Y-CD: Conceptualization, Funding acquisition, Investigation, Project administration, Resources, Supervision, Writing – review & editing.
