# Supplementary material for: Serological surveillance of GI norovirus reveals persistence of blockade antibody in a Jidong community-based prospective cohort, 2014–2018
Source: Front Cell Infect Microbiol. 2023 Dec 18;13:1258550. doi: 10.3389/fcimb.2023.1258550 (PMC10766831; doi:10.3389/fcimb.2023.1258550)
Supplement: Supplementary file 1 [file DataSheet_1.pdf]

Supplementary Table S1: Seroreversion rates of blockade antibodies against GI NoV in Jidong community-based cohort, 2014-2018

Observation year	GI.2		GI.3		GI.9	
	Seroreversion %（n/N）	*P* value	Seroreversion %（n/N）	*P* value	Seroreversion %（n/N）	*P* value
**Total**	84.8%（55/66）		54.8（57/104）		59.7%（43/72）	
**2015**	50.0%（33/66）	**0.031***	16.3%（17/104）	**<0.001*****	34.7%（25/72）	**0.009****
**2016**	39.0%（13/33）		9.2%（8/87）		12.8%（6/47）	
**2017**	25.0%（5/20）		8.9%（7/79）		12.2%（5/41）	
**2018**	33.0%（5/15）		34.7%（25/72）		19.4%（7/36）	

*P<0.05, **P<0.01, ***P<0.001.
